# Health-related quality of life and psychological status of women with primary Sjögren's syndrome: A cross-sectional study of 304 Chinese patients

**DOI:** 10.1097/MD.0000000000009208

**Published:** 2017-12-15

**Authors:** Zhaoxiang Liu, Zhenhua Dong, Xiaochun Liang, Jinhe Liu, Lei Xuan, Jing Wang, Gaili Zhang, Weixin Hao

**Affiliations:** a Department of Traditional Chinese Medicine, Translational Medicine Center, Peking Union Medical College Hospital, Chinese Academy of Medical Science; b Beijing Tsinghua Changgung Hospital, School of Clinical Medicine, Tsinghua University, Beijing, China.

**Keywords:** anxiety, depression, Hospital Anxiety and Depression Scale, primary Sjögren syndrome, quality of life, Short Form (36) Health Survey

## Abstract

Patients with primary Sjögren syndrome (pSS) always suffer from dryness, pain, and fatigue caused by the involvement of multiple different systems or organs. The uncomfortable disease symptoms, the consequent disability, and the side effects of therapeutic drugs decrease the quality of life and lead to emotional problems. We investigated the health-related quality of life and psychological status of a large cohort of women patients with pSS and associated factors.

A total of 304 women with pSS referred to Peking Union Medical College Hospital during 2011 and 2014 were included. The internationally recognized Short Form (36) Health Survey (SF-36) was used to assess patients’ quality of life; a higher score indicated a better quality of life. Patients’ psychological status was assessed by the Hospital Anxiety and Depression Scale (HADS), and higher scores predicted more anxiety or depression.

Patients with pSS had remarkably lower SF-36 scores. The Hospital Anxiety Scale (HAS) and Hospital Depression Scale (HDS) scores of the pSS patients (7 [4,10] and 6 [3,10], respectively) were significantly higher than that of patients with other internal diseases (3.37 ± 2.81 and 3.83 ± 3.14; both *P* < .001). Negative predictors of quality of life were: pain (physical condition, β = –0.225; *P* < .001); fatigue (physical condition, β = –0.298; *P* < .001; and mental condition, β = –0.319; *P* < .001). Risk factors for anxiety were: young age (β = –0.059; *P* = .035); pain (β = 0.025; *P* = .028); or fatigue (β = 0.029; *P* = .004). Risk factors for depression were: xeroderma (β = 0.030; *P* = .003); pain (β = 0.022; *P* = .047); or fatigue (β = 0.033; *P* = .001).

Patients with pSS have a low quality of life with anxiety and depression. Pain and fatigue are primary factors for lower quality of life, which cause more anxiety and depression.

## 1 Introduction

Sjögren's syndrome (SS), caused by the attack of immune cells on the exocrine glands, is a systemic autoimmune disease characterized by progressive xerostomia and xerophthalmia. Besides the damage to exocrine glands, patients with SS can also suffer from arthritis, myalgia, skin, and changes in the functions of multiple systems or organs^[1]^; serum autoantibodies and immunoglobulins are usually elevated. Primary Sjögren syndrome (pSS) is a subgroup of SS, in which a concurrent secondary connective tissue disease is absent. When multiple systems or organs are involved, a combination of steroids and immunosuppressive drugs is used to control the disease. However, these drugs can cause serious side effects. Thus, the quality of life of pSS patients is substantially degraded by the uncomfortable symptoms of the disease, the consequent disability, and the side effects of therapeutic drugs. This physical suffering in turn causes various degrees of mental stress from panic, anxiety, depression, sadness, and other emotional problems,^[2,3]^ which further negatively affect patients’ physical functions and quality of life.

Patients with Sjögren syndrome (SS) may be restricted in their activities and social participation. This results in a reduced health-related quality of life and devastated socioeconomic status (due to lower employment rates and more disability). Hence, coping, social support, optimism, and life satisfaction are significant components of the health-illness continuum. Therefore, more attention should be paid and care provided to those patients.

In this study, we evaluated the quality of life and mental health status of a large cohort of women Chinese patients with pSS (n = 304). In addition, we analyzed the primary factors contributing to lower quality of life and increased anxiety and depression, providing evidence and solutions to the situation.

## 2 Methods

### 2.1 Study design

From January 2011 to December 2014, 396 women pSS patients were invited to participate in this investigation. The Medical Outcomes Study (MOS), 36-item short form health survey (SF-36), and Hospital Anxiety and Depression Scale (HADS) were used to evaluate patients’ health-related quality of life and psychological status, respectively. Only 304 patients consented and completed the questionnaire. All subjects joined the study voluntarily after signing prior informed consent forms. The study protocol was reviewed and approved by the Ethics Committee of the Peking Union Medical College Hospital, Peking, China (ethical approval number: S-K076).

As no large-scale general SF-36 population survey related to quality of life has been conducted in China, we employed the assumption that the SF-36 study of 17,754 people from 6 provinces in China presented the general population. To understand the extent of the lower quality of life, we compared the quality of life of 309 pSS women to that of general people.^[4]^

Moreover, no large-scale HADS survey exists on the psychological status of the general Chinese population. In theory, people who are being treated for multiple other diseases (not for anxiety and depression) in the Internal Medicine Department would suffer from anxiety and depression. We compared HADS of pSS patients to that of the participants (n = 6581) treated in the Internal Medicine Department to illustrate indirectly the severity of psychological status of pSS patients.^[5]^

Diagnosis of pSS was made in accordance with the classification (diagnostic) criteria approved by the American College of Rheumatology (ACR) (2012). The criteria were ≥2 of the following 3 criteria^[6]^: the detection of serum anti-Ro/SSA (i.e., anti-SS-related antigen A) or anti-La/SSB (anti-SS-related antigen B antibodies, or positive rheumatoid factor and antinuclear antibody; a positive salivary gland biopsy exhibiting a focal lymphocytic sialadenitis with a focus score ≥1; or the presence of keratoconjunctivitis sicca. The age range of all patients was from 18 to 70 years. Patients with diabetes, malignant tumors, and severe organ diseases or disabilities not caused by pSS were excluded.

### 2.2 Items and methods of observation

The internationally recognized health status survey questionnaire (the Short Form [36] Health Survey, or SF-36^[7]^ and the Hospital Anxiety and Depression Scale (HADS)^[8]^ were used to investigate the pSS patients’ quality of life and psychological status. Patients’ clinical presentation, systems involvement, and co-morbidities were recorded. Self-reported (SF) severity of pain, fatigue, and dryness were judged by visual analogue scale (VAS, scored 0–10).

### 2.3 SF-36

SF-36, which has been documented to have acceptable reliability and validity in a Chinese population, is used widely to evaluate people's health-related quality of life.^[9]^ It includes 8 items: physical functioning; role limitations due to physical health problems; pain; perceptions of general health; vitality; social functioning; role limitations due to emotional problems; and mental health. The score ranges from 0 to 100, with 0 indicating the worst and 100 representing the best condition. Thus, a higher score indicates a better quality of life. The 8 items were divided to obtain scores for physical condition (PCS) and mental condition (MCS). Specifically the PCS averaged the results for the following items: physical functioning; role limitations due to physical health problems; pain; and perception of general health. Similarly, the MCS averaged the results for: vitality; social functioning; role limitations due to emotional problems; and mental health.

### 2.4 HADS

The HADS included 14 questions, 7 of which were associated with depression, and the other 7 were related to anxiety. Each question was scored from 0 to 3 points. If the total score for anxiety or depression was >7, mental symptoms were suspected in these patients. If the scores were >9, patients were considered anxious or depressed.

### 2.5 VAS

The severity of symptoms was graded at 10 levels, with zero representing the absence of pain and 10 denoting the worst pain possible. All patients were asked to self-assess different symptoms.

### 2.6 Statistical analysis

SPSS version 19.0 software was used for data analysis. Normally distributed data are expressed as the mean ± standard deviation. Data that were not normally distributed are reported as median (quartile). To compare the patient and control groups, the independent sample *t* test or nonparametric test was used based on the numerical properties. The correlation between every 2 variables was analyzed according to Spearman's rank correlation. A multivariate linear regression model was built to identify the factors that influence quality of life and psychological status. The following were considered independent variables: age; disease course; educational level; family history of connective tissue disease; co-morbidities; systems or organs involved; pain; fatigue; xerostomia; xerophthalmia; xeromycteria; xeroderma; tracheal dryness; colpoxerosis score; and therapeutic regimen. Scores for the following independent variables were taken by VAS: pain; fatigue; xerostomia; xerophthalmia; xeromycteria; xeroderma; tracheal dryness; and colpoxerosis. The therapeutic regimen, as an independent variable, was scored as: 0, untreated; 1, treated with Traditional Chinese Medicine; 2, treated with Western medicine; and 3, treated with combined Western and Traditional Chinese Medicine. Statistical significance was set at *P* < .05.

## 3 Results

### 3.1 Clinical characteristics of pSS patients

A total of 304 women with pSS who completed all the questionnaires were included in the investigation. The mean age of the patients was 49 years (range: 40–56 years). The average course of the disease was 5 years (range: 3–10 years). Of all the patients, 50% (152/304) had obtained an educational level higher than high school. Forty-one patients (13%) had a family history of connective tissue diseases. Disease severity was self-reported as mild, moderate, or serious, and most of the patients (47%) selected the moderate status. Approximately 31% of the patients thought the severity of disease they experienced was best classified as mild, whereas 22% identified theirs as serious.

Seventeen patients had been untreated since the diagnosis had been made. Nearly 50% of the patients (144/304) were treated with combined Western and Traditional Chinese Medicine. Ninety-seven patients received Traditional Chinese Medicine only, and 42 received Western medicine only. Almost 64% of the patients had other, accompanying internal diseases.

The involvement of organs or systems was common in pSS patients. More than 90% of the patients were found to have damage in multiple organs or systems, including the hematologic, respiratory, gastrointestinal, urinary, nervous, endocrine, skin and mucosa, and joints and muscles.

### 3.2 Quality of Life of pSS patients

For the 304 patients, the results of the SF-36 questionnaires showed a mean PCS score of 55.72 ± 19.78, and mean MCS score of 50.81 ± 22.91 after SF-36. Compared with the general population in 6 Chinese provinces,^[4]^ the quality of life of patients with pSS was significantly poorer in all dimensions outlined in the SF-36, and the differences were significant (*P* < .001; Table 1).

**Table 1:**
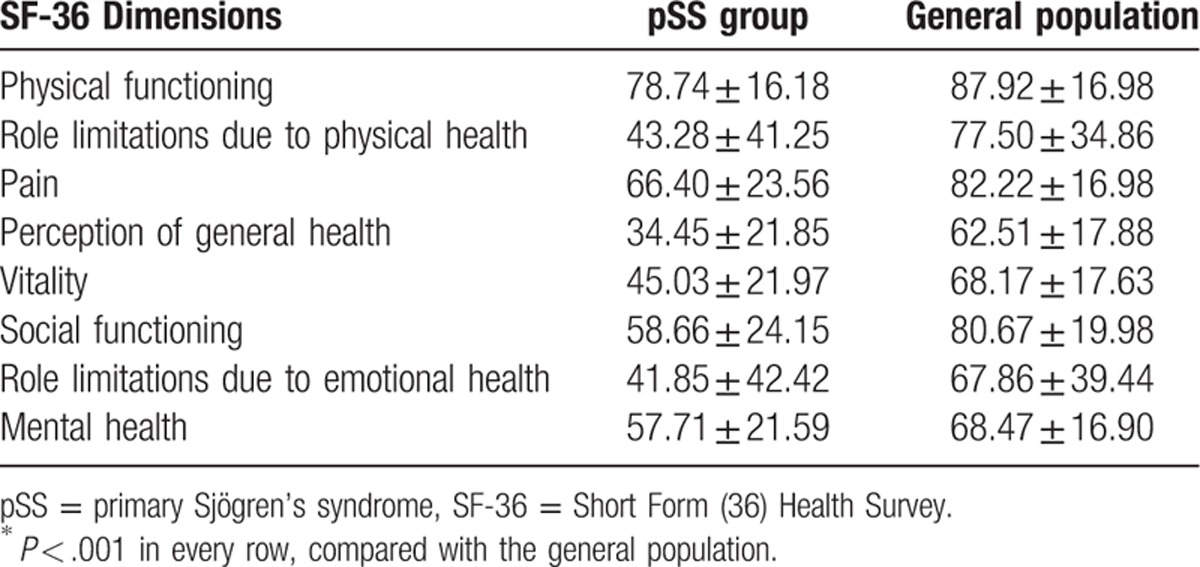
SF-36 dimensions of pSS patients and the general population; scores^∗^.

### 3.3 Factors influencing the quality of life in pSS patients

The PCS and MCS scores were calculated separately as dependent variables. Multiple regression analysis showed that pain (β = –0.225, *P* < .001) and fatigue (β = –0.298, *P* < .001) were associated with decline of PCS (*P* < .05). Fatigue (β = –0.319, *P* < .001) was associated with a decrease of MCS (Table 2).

**Table 2:**
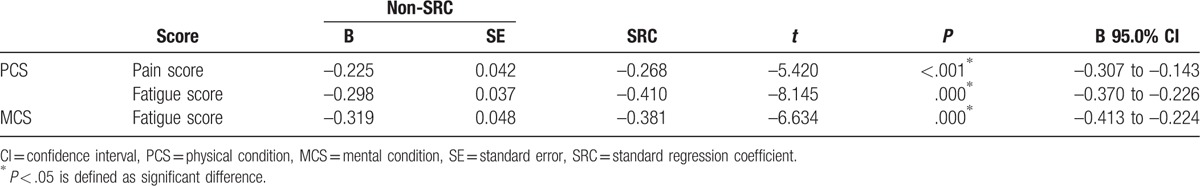
Primary factors contributing to PCS and MCS (multivariate linear model analysis).

### 3.4 Psychological status of pSS patients

The data from our analysis showed that the 304 female pSS patients scored 7^[4,10]^ on the Hospital Anxiety Scale (HAS); 129 (42.4%, 129/304) pSS patients obtained a HAS score >7, and 86 patients (28.3%, 86/304) had a HAS score >9. All patients scored 6^[3,10]^ in HDS, whereas 126 patients obtained a Hospital Depression Scale (HDS) score higher than 7 (40.78%), and in 79 patients, it was higher than 9 (25.57%). When compared to the HADS score of patients treated in the Internal Medicine Departments in Shanghai (n = 11,766),^[5]^ the HAS and HDS scores of the pSS patients in the present study were considerably higher than those of patients suffering from other internal diseases [HAS, 7^[4,10]^ cf. 3.37 ± 2.81, *P* < .001; HDS, 6^[3,10]^ cf. 3.83 ± 3.14, *P* < .001].

### 3.5 Factors influencing anxiety and depression in pSS patients

HAS and HDS were set as dependent variables separately. Multiple regression analysis revealed that age (β = –0.059, *P* = .035) was related to the decline of HAS score, but pain (β = 0.025, *P* = .028) and fatigue levels (β = 0.029, *P* = .004) were associated with an increase in HAS score (Table 3). Pain (β = 0.022, *P* = .047), fatigue (β = 0.033, *P* = .001), and xeroderma scores (β = 0.030, *P* = .003) were all related to the increase in HDS (Table 3).

**Table 3:**
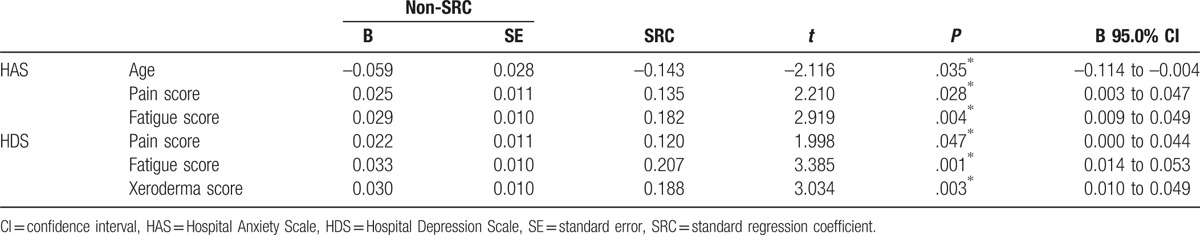
Primary factors contributing to HAS and HDS (multivariate linear model analysis).

### 3.6 Correlation between quality of life and HADS score

The correlations between PCS/MCS and HAS/HDS were analyzed separately via Spearman's correlation coefficient. The results indicated that PCS and MCS correlated negatively with HAD (*P* < .001) and HDS (*P* < .001).

## 4 Discussion

To analyze the quality of life and psychological status of pSS patients and identify the relevant associated factors in a large cohort of pSS patients, we conducted a cross-sectional study of 304 women patients referred to our hospital. Most of the patients were from 50 to 59 years old, and reported that symptoms of pSS first occurred at ages from 40 to 60 years, which was in accordance with the published characteristics of the disease.^[10]^ Our data revealed low quality of life and high anxiety and depression scores in our pSS patients, which is consistent with other reports.^[3,11]^

Studies have shown that pSS patients suffer from long-term fatigue and pain in the muscles and joints.^[12,13]^ The results of the multiple regression analysis of the present investigation indicated that pain and fatigue were the primary contributors to decline in quality of life. Segal et al^[14]^ indicated that serious fatigue, pain, and older age were the primary influencing factors of a lower quality of life. Meijer et al^[15]^ concluded that fatigue, tendomyalgia, comorbidity, male gender, and receiving disability compensation were related to the decline of PCS according to the SF-36 scale, while fatigue, articular involvement, use of artificial saliva, use of antidepressants, and comorbidity were related to decline of MCS. Our present investigation revealed that pain and fatigue were factors influencing PCS, whereas fatigue was the factor influencing MCS, which was consistent with Segal's results. Fatigue and pain limit patients’ physical activity and social life, consequently affecting their overall quality of life. Therefore, clinical treatment for pSS should focus on pain and fatigue.

Generally, xerosis affects digestion and daily speech and also leads to various oral diseases, which consequently impair the physical function and mental status of the affected patients. In the present work, we did not find a correlation between xerosis symptoms and decline of quality of life. Stewart et al^[16]^ and Enger et al^[17]^ established that oral conditions significantly impaired the quality of life of patients with pSS and stressed the importance of improving the oral condition. Belenguer et al^[18]^ concluded that colpoxerosis (in women) may be related to decline of quality of life. However, Champey et al^[19]^ failed to find any correlation between xerosis symptoms and quality of life. The discrepancy between the findings of these studies may be caused by different scales, sampling error, or cultural differences. Patients in China may have insufficient knowledge about SS, and poor treatment outcomes, long disease course, and misunderstanding of the disease may be other contributors to the decline in quality of life.

The present study revealed that HAS and HDS scores of pSS patients were significantly higher (indicating obvious anxiety and depression) than those of patients treated in the Internal Medicine Departments in Shanghai. The multiple regression analysis results indicated that the age of patients negatively correlated with anxiety. That is, the younger the patient, the greater the anxiety. Pain and fatigue levels were also related to anxiety, whereas pain, fatigue, and xeroderma were associated with depression.

Multiple investigations and observations have shown that the prevalence of anxiety and depression is significantly higher in patients with pSS than in the general population. Both Valtysdottir et al^[20]^ and Stevenson et al^[21]^ adopted HADS to assess pSS patients’ psychological status. The patients had higher scores for anxiety and depression than the control group and a higher risk of developing clinical depression than rheumatoid arthritis. Bax et al^[22]^ and Barendregt et al^[23]^ concluded that depression in patients with SS positively correlated with fatigue. Chen et al^[24]^ completed a retrospective analysis of 31 patients admitted to the hospital for mental disorders induced by SS; 23 patients were admitted to a psychiatric department for the first time before their diagnosis of pSS was confirmed, and 5 patients were hospitalized several times in a psychiatric department. Before the confirmation of SS, the major psychiatric manifestations were anxiety and depression.

Our present study confirmed that patients with pSS are more susceptible to anxiety and depression than other patients. This phenomenon may be attributable to the factors discussed below. First, some scholars suppose that emotional disorders in patients with SS may be a sign of central nervous system involvement. Mataro et al^[25]^ and Segal et al^[26]^ performed brain-imaging examinations of SS patients and discovered cerebral structural abnormalities associated with cognitive impairment, concluding that the brain organic changes in patients with SS are related to their cognitive function change.

Second, there is evidence that patients with anxiety and depression experience immunity activation and the release of cytokines.^[27]^ The immunologic derangement and long-term increase of pro-inflammatory cytokines in patients with pSS may change the metabolites of the neuroendocrine and central nervous system, causing or aggravating anxiety and depression. For example, Xie et al^[28]^ found that the number of purinergic 2 X 7 (P2X7) receptors expressed on peripheral blood mononuclear cells in pSS patients were significantly higher than that of the controls, suggesting that in pSS patients P2X7 receptors may contribute to anxiety, depression, or both.

Third, many patients with pSS complain of fatigue and joint pain, symptoms proven in this study to be the major contributing factors to anxiety and depression. Xerosis, the most common clinical feature of pSS, may also contribute to anxiety and depression.

Fourth, our data showed that younger patients were more likely to be anxious. One possible explanation may be that they have a higher standard for quality of life and pay more attention to the disease than older patients do.

Finally, for most female pSS patients, the disease occurs during the perimenopausal period when women are apt to experience mood swings. It is also noted that a family burden and economic pressures aggravate anxiety and depression.

Champey et al^[19]^ studied the quality of life, anxiety, and depression of 111 patients with pSS. The results revealed that the scores in the physical and psychological sections of the SF-36 were significantly related to the overall symptom index in the symptom checklist (SCL-90, 1 type of Rating Scale for Mental Health), stressing the importance of mental dimensions in SF-36. The PCS and MCS in the present study showed a negative correlation with HAD and HDS, which meant that decline in the quality of life was significantly related to anxiety and depression. Anxiety and depression appeared to exert a negative effect not only in the psychological dimension but also in the physical. Therefore, early detection and proper intervention of anxiety and depression in pSS patients is important to improve their quality of life.

There are several limitations in our study. First, the inherent nature of a cross-sectional investigation was the main shortcoming. Prospectively designed, multiple-centered examinations are needed to validate these results. Second, we did not analyze data from male pSS patients due to their low number in the population.

## 5 Conclusion

In the present study, we confirmed that a perception of low quality of life with anxiety and depression was present in a large cohort of women Chinese pSS patients. Pain and fatigue were the primary contributing factors to lower quality of life and increased anxiety and depression. On the other hand, delayed diagnosis, protracted course of the disease, and the lack of social support are also causes of the decline in the quality of life in these patients.

## 6 Relevance to clinical practice

Progressive xerostomia and xerophthalmia are always manifested in pSS patients. Some of them also suffer from arthritis, myalgia, skin, and multiple involvements of systems or organs. This unpleasant experience, along with the effect of the disease, may aggravate the psychological burden and induce various degrees of negative emotions, increasing the risks of developing clinical depression, which in turn exacerbate decline of the quality of life. The findings from the present work indicate that we should pay more attention to pSS patients’ quality of life and psychological status. Alleviating pain and fatigue would be beneficial to obtaining better quality of life. Psychological counseling and support are also extremely important to those patients.

Many measures can be carried out to improve pSS patients’ quality of life and psychological status. First, more special training of practitioners and nurses is needed to offer early and effective treatment of patients. Second, courses or training sessions to educate patients could help them deepen their understanding and knowledge about the disease. Third, more publicity and health care provided by the government could be of considerable importance to the improvement of pSS patients’ quality of life.
